# Birds, primates, and spoken language origins: behavioral phenotypes and neurobiological substrates

**DOI:** 10.3389/fnevo.2012.00012

**Published:** 2012-08-16

**Authors:** Christopher I. Petkov, Erich D. Jarvis

**Affiliations:** ^1^Institute of Neuroscience, Newcastle UniversityNewcastle upon Tyne, UK; ^2^Centre for Behavior and Evolution, Newcastle UniversityNewcastle upon Tyne, UK; ^3^Department of Neurobiology, Howard Hughes Medical Institute, Duke UniversityDurham, NC, USA

**Keywords:** evolution, humans, monkeys, avian, vertebrates, communication, speech, neurobiology

## Abstract

Vocal learners such as humans and songbirds can learn to produce elaborate patterns of structurally organized vocalizations, whereas many other vertebrates such as non-human primates and most other bird groups either cannot or do so to a very limited degree. To explain the similarities among humans and vocal-learning birds and the differences with other species, various theories have been proposed. One set of theories are motor theories, which underscore the role of the motor system as an evolutionary substrate for vocal production learning. For instance, the motor theory of speech and song perception proposes enhanced auditory perceptual learning of speech in humans and song in birds, which suggests a considerable level of neurobiological specialization. Another, a motor theory of vocal learning origin, proposes that the brain pathways that control the learning and production of song and speech were derived from adjacent motor brain pathways. Another set of theories are cognitive theories, which address the interface between cognition and the auditory-vocal domains to support language learning in humans. Here we critically review the behavioral and neurobiological evidence for parallels and differences between the so-called vocal learners and vocal non-learners in the context of motor and cognitive theories. In doing so, we note that behaviorally vocal-production learning abilities are more distributed than categorical, as are the auditory-learning abilities of animals. We propose testable hypotheses on the extent of the specializations and cross-species correspondences suggested by motor and cognitive theories. We believe that determining how spoken language evolved is likely to become clearer with concerted efforts in testing comparative data from many non-human animal species.

## Introduction

Charles Darwin's theory on descent with modification as it applies to man (Darwin, 1871) had for many years been used to underscore the importance of non-human primates for unraveling the origins and neuronal precursors of spoken language (e.g., Hewes, 1973). Yet, in part because of the apparent lack of vocal learning or syntactic-like abilities in non-human primates, different camps have focused on either the differences between human and non-human primates or their similarities. This has resulted in many contentious debates on language evolution with regards to non-human primates (for reviews: Pinker, 1994; Hauser et al., 2002; Pinker and Jackendoff, 2005). Adding further complexity for understanding spoken language origins, recently the research focus has shifted towards species more distantly related to humans, such as certain groups of songbirds. This is in part because songbirds like humans and a few other species exhibit vocal learning and have what has been broadly classified as “syntactic-like” song production (Doupe and Kuhl, 1999; Jarvis, 2004; Bolhuis et al., 2010). A summary of a consortium on the origins of human language syntax and its biological foundations encapsulates some of the current thinking:

Another area of agreement might seem surprising in light of many current “primate-centric” studies of language evolution (Burling, 2006; Hurford, 2007). Most participants felt that there were no true precursors of syntax to be found among our nearest relatives. For anything like a syntactic precursor one had to go as far afield as songbirds ….

(Bickerton and Szathmary, 2009)

Likewise, in a thought provoking essay, Bolhuis and Wynne (2009) questioned to what extent evolutionary theory can help us to understand cognitive brain mechanisms in living animals. Their perspective was illustrated by a cartoon depicting a scientist with the great hope of teaching a monkey to say “apple,” but realizing that the monkey is the classroom dunce when the parrot vocally identifies the apple variety as “golden delicious.” Darwin, however, would have likely filled the classroom with as many different animals as possible. In any case, the authors' conclusions are appropriately nuanced and seem to favor a broader comparative approach: “there is no a priori reason to assume that convergence will be more important than common descent or vice versa” (Bolhuis and Wynne, 2009).

We, as researchers that have studied non-human primates and birds, argue that the path toward understanding the origins of spoken language cannot be based on focusing on a few select species with or without communication abilities that are either thought to be most comparable to humans, or to reflect physiology most comparable to humans. Any “one animal centric” approach will only limit our capacity to unravel the evolutionary bases of spoken language. If for no other reason, without “other” species as points of reference, it would not be clear what is special about human communication. Moreover, a focus on certain species restrains the development of different animal model systems with distinct advantages for understanding the neurobiological mechanisms of human language-related processes, which is important for advancing treatment options for communication and language disorders. Thus, to better understand the origins of human spoken language we rely on a broad comparative approach that takes advantage of information obtained across animal taxa, letting each animal have their “say” on the question of language evolution. We are aware that to do so can only be achieved by additional comparative work that will require energy and investment, combined with efforts to stay objective, as best as we can, regarding the cross-species similarities and differences.

In an effort to invigorate a broader perspective on spoken language origins, in this paper we overview the parallels and differences in the behavioral and neurobiological data of vocal learners (e.g., humans and songbirds) and those animals often identified as “vocal non-learners.” We ask how strong is the evidence for categorical distinctions between vocal learners and vocal non-learners? We note that vocal non-learners are often classified as such based on a lack of experimental evidence, but that when the animals are tested, there is often more variation in vocal learning abilities than might have been expected (Janik and Slater, 2000; Snowdon, 2009; Arriaga et al., in press). Moreover, since vocal learning depends on auditory learning, and auditory learning abilities are broadly conserved in the animal kingdom, we ask how this trait dependency could have influenced the evolution and mechanisms of vocal learning. Then, based on a modified perspective of the literature we reconsider some of the motor and other theories that have been proposed for humans, birds and other animals. We conclude by generating testable hypothesis, including for: (1) better understanding variability in the vocal behavior and neurobiology of vertebrates that are often classified as vocal non-learners; and (2) the possible capabilities of, for example, non-human primates as limited vocal learners but considerable auditory learners, to learn the structure of auditory sequences, and whether this might tap into an ancestral “proto-syntactic” brain network that evolved in humans to support syntactic learning.

## Vocal production learning and auditory learning: how are these behavioral phenotypes distributed?

**Figure 1:**
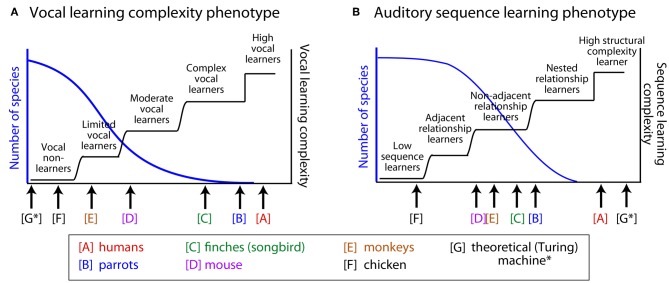
**Hypothetical distributions of two behavioral phenotypes: vocal learning and sensory (auditory) sequence learning.** We hypothesize that the behavioral phenotypes of vocal learning and auditory learning are distributed along several categories. **(A)** Vocal learning complexity phenotype and **(B)** auditory sequence learning phenotype. The left axis (blue) illustrates the hypothetical distribution of species along the behavioral phenotype dimensions. The right axis (black step functions) illustrates different types of transitions along the hypothesized vocal-learning **(A)** or auditory-learning **(B)** complexity dimensions. See manuscript text for the basis for the relative position of the non-human animals illustrated in this figure, which in some cases is based on limited data. Also see Arriaga and Jarvis (in press) for an initial proposal of this idea. Whether the actual distributions are continuous functions (blue curves), will need to be tested, in relation to the alternatives that there are several categories with gradual transitions or step functions (black curves). Although auditory learning is a prerequisite for vocal learning and there can be a correlation between the two phenotypes (A–B), the two need not be interdependent. A theoretical Turing machine (Turing, 1968) is illustrated [G^*^], which can outperform humans on memory for digitized auditory input but is not a vocal learner.

Behavioral data demonstrating that an animal can learn to produce novel vocalizations is often used to classify different species as either vocal learners or vocal non-learners (Nottebohm, 1976; Janik and Slater, 1997; Jarvis, 2004). However, once some animals within a taxonomic group are characterized as vocal learners, we cannot assume that all animals of that group have vocal production learning abilities to the same degree. For instance, different song learning birds have different levels of complexity in their song production, and humans (including infants) can be regarded as exceptional vocal learners (i.e., *high-end* of *vocal learners*, see Figure 1). Among passerine songbirds, some species learn to produce only one song that was learned early in life, while others can learn many songs with some level of continuous learning throughout adulthood (Catchpole and Slater, 1995; Okanoya, 2004). For example, songbirds such as zebra finches tend to learn one song type as juveniles. Such songs often have strictly-linear transitions that step through the different song syllables in a motif from beginning to end (Honda and Okanoya, 1999). On the other hand, the songs of mockingbirds, nightingales and humpback whales show considerably greater variability. Some of these song elaborations show repetitions of particular elements within a range of legal repetitions and can include forward or backward branching relationships in how the animals transition between the different elements of their song, as well as non-adjacent relationships between distant song elements. Such “syntactic-like” structure in songbirds has drawn the interest of linguists and cognitive neuroscientists (e.g., Bickerton and Szathmary, 2009; Berwick et al., 2011; Hurford, 2012).

Moreover, not all vocal learners are known to be able to imitate the vocalizations of other species, called vocal mimicry. Yet, one of the initial tests that some have used as evidence to conclude that non-human primates are not vocal learners was the difficulty that chimpanzees have in imitating human speech (e.g., Rumbaugh, 1977; Shettleworth, 2010). Some animals such as corvid songbirds (e.g., crows, jays and magpies) and African Grey and Amazon parrots are exceptional imitators that can imitate human speech (e.g., Kroodsma, 1976; Pepperberg, 2010). The birds at this end of the spectrum are fairly easy to identify since they often imitate without training or an obvious reward. Moore (2004) notes that, “Moore's (1992) parrot, for example, repeatedly mimicked a swear word that it had heard only once, through a closed door.”

Another important issue is that the distinction between vocal learning and non-learning captures only the more apparent differences in vocal production behavior. Song learning in birds and speech learning in humans takes at least two phases: auditory learning and sensory-motor vocal learning (Doupe and Kuhl, 1999). Only the latter is evident in vocal production behavior. For example, many of us are familiar with the situation where as we learn a second language we progress through stages where our ability to understand the language outstrips our ability to produce it. Thereby, in discussing how the human brain has specialized to support spoken language, not only do we need to consider the cross-species variability of specific behavioral phenotypes, but we also need to distinguish different behavioral phenotypes, such as sensory (auditory) learning and vocal production learning (Jarvis, 2004).

In this section, we distinguish between vocal production and auditory learning abilities and consider how these two behaviors might be distributed. Why does the variance in a behavioral phenotype matter? It is important to determine the ways in which, for example, vocal learning is distributed since each possibility carries with it different implications for: (1) how spoken language is likely to have originated; (2) the evolutionary pressures that regulate the presence and absence of a behavioral phenotype; and, (3) whether few or many animals might serve to model certain mechanistic aspects of human speech- and language-related processes.

We note that when the findings of recent studies are examined across species, there seems to be evidence for greater variability in the experience-based ability to modify vocalizations. This variability is greater than would be expected in animals often assumed to be vocal non-learners (e.g., Saranathan et al., 2007; Snowdon, 2009; Briefer and McElligott, 2011; Arriaga et al., in press). Our review of the literature suggests that the currently known vocal learners could be more accurately described as *complex-vocal leaners* (humans potentially different among these as *high vocal learners*), and other species as *moderate-vocal learners*, *limited-vocal learners*, or obligate *vocal non-learners*. Thereby, the empirical evidence does not fit well with a binary categorical distinction between vocal learning and non-learning. Instead, the evidence better fits with the notion of a recently proposed vocal-learning continuum hypothesis (Arriaga and Jarvis, in press). We conclude by considering how approaches in linguistics can be useful for unraveling the complexity of animals' vocal production and/or auditory learning capabilities.

### Variability in the vocal learning phenotype

We begin with the useful designations of vocal learning made by Janik and Slater (1997), but with our modifications of their three categories as: production vocal learning; usage vocal learning; and, auditory learning (instead of comprehension learning).

*Production vocal learning* is often defined as the ability of an animal to produce novel vocalizations. There are various ways in which this can be tested. These include: (1) cross-fostering experiments, such as when an infant can learn the vocalizations of surrogate parents of the same or other species; (2) experiments evaluating changes in vocalizations in response to different types of competing sounds; (3) social isolation studies where the animal does not have access to a model to imitate; and (4) deafening experiments where the animal cannot hear others or itself. The strongest evidence that vocal production learning has occurred is demonstrating that an animal can imitate vocalizations of other species or sounds. Vocal imitation depends upon the animal being able to hear and to have a model to imitate. As such, deaf *vocal learners* usually show acoustically degraded, species non-typical sounds, whereas deaf *vocal non-learners* typically show vocal production behavior that does not differ from wild type animals. Sometimes an argument is made that the effects of social isolation or deafening could be due to unspecified impacts on behavior. This becomes less of an issue if it is shown that the behavioral impact is specific to the vocal learning modality.

Another careful distinction that needs to be made is defining the anatomical source of the “vocalizations” that are found to be learned. Sounds generated by the vocal organ (larynx in mammals; syrinx in birds) are in the strictest sense, vocalizations, whereas those generated by lips, teeth, and tongue are non-voiced, oro-facially generated or modified sounds. The neural mechanisms and the degree of control of the different musculature for generating voiced or unvoiced sounds could differ. Whatever the approach, to substantiate that an animal is capable of production vocal learning one needs to document a convincing experience-dependent change in vocal production behavior, with consideration of the source of the sounds.

*Usage vocal learning* is when an animal learns to use acoustically innate or already learned vocalizations in a new context. Although usage vocal learning involves the learning of the contexts in which to vocalize, it is not production vocal learning because it does not require modification of the acoustic structure of a vocalization to create one that the animal did not have in its repertoire. Common examples of usage vocal learning are the predator alarm calls of vervet monkeys and chickens, where juveniles learn through social experience the context within which to generate the innate call or the appropriate behavioral response (e.g., descend from trees, tilt head, etc.) upon hearing the call from a conspecific (Seyfarth et al., 1980; Evans et al., 1993; Snowdon, 2009).

*Auditory learning* is when an animal learns to perceive something novel or behaviorally react to sounds differently as a result of experience. For example, a dog learns how to associate the human sounds “sit” or “sientese” with the act of sitting, but it does not learn how to produce or vocally use these sounds. Even with this capacity, there might be limits to the complexity of verbal commands that an animal might be able to learn in the auditory modality (Moore, 2004).

#### Who are the production vocal learners?

All vocal species tested appear to have varying degrees of auditory learning and usage vocal learning (Moore, 2004; Schusterman, 2008), but only relatively few have production vocal learning (Janik and Slater, 1997; Jarvis, 2004). The later include, in addition to humans, three groups of birds (passerine songbirds, parrots and hummingbirds; Marler and Tamura, 1964; Jarvis, 2004; Jarvis et al., 2005; Bolhuis et al., 2010; Pepperberg, 2010), some species of bats (Esser, 1994; Boughman, 1998), and pinnipeds and cetaceans (Noad et al., 2000). For example, in several cases, a harbor seal and dolphin were found to imitate human vocalizations (Lilly, 1965; Ralls et al., 1985). This list has recently expanded to include elephants (Poole et al., 2005), where an African elephant was shown to imitate Asian elephant calls and another elephant to imitate the sounds of passing trucks. In the rest of this paper we will refer to these species as vocal learners, meaning production vocal learners.

#### Who are the vocal non-learners?

The answer to this question is much less clear. In contrast to the few known vocal learning species, it is commonly thought that most other vertebrates are not capable of vocal imitation: that is, they are not capable of the type of learning that leads to the production of novel communication signals not within their repertoire or to the production of acoustic changes to innate vocalizations. However, most of these animals have not been formally or rigorously tested to determine whether they have more limited capabilities for some aspects of production vocal learning. That is, many vertebrates are usually placed in the “vocal non-learning” category based largely on a lack of evidence rather than evidence for a lack of any vocal learning capabilities. In the last few decades, with the use of refined acoustical analysis tools and hypothesis-driven experimental strategies, evidence appears to be accumulating that some of the, so-called, vocal non-learners have measurable levels of vocal flexibility to change some of the acoustics in their vocalizations, potentially a limited form of vocal learning. We will consider several examples of this from work in non-human primates (Snowdon, 2009), birds (Saranathan et al., 2007), rodents (Arriaga et al., in press), and goats (Briefer and McElligott, 2011).

For non-human primates, the earlier studies are difficult to interpret, in part because of contradictory conclusions. One study reported what seemed like ontogenetic acoustical changes to innate vocalizations in a cross-fostering study of two species of macaques (Masataka and Fujita, 1989). This finding was later challenged by lack of independent replication of the findings and on technical grounds (Owren et al., 1992). For a review see (Egnor and Hauser, 2004). Furthermore, many of the more striking examples of non-human primates having changed their vocalizations have been shown between regionally separated animals, which could be based primarily on genetically regulated differences between populations (Snowdon, 2009). More recently, a number of studies have shown that non-human primates can make some limited modifications to their presumably innate vocalizations via laryngeal control. For instance, there are several examples of developmental maturation of vocalizations toward their adult form that might not be entirely consistent with innate developmental changes in the vocal production apparatus (e.g., vervets: Seyfarth and Cheney, 1986; prosimians: Zimmerman, 1989; squirrel monkeys: Hammerschmidt et al., 2001). Additional evidence that could question the “vocal non-learning” label in non-human primates has been from call convergence studies, where either two marmosets or macaques housed together for a few weeks showed convergence in the pitch and some other acoustic features of innately determined calls (reviewed in: Snowdon, 2009). Also, there is evidence that adult Japanese macaques are able to adjust the fundamental frequency of their innately-specified vocalizations to match playbacks presented to them of different conspecifics (Sugiura, 1998).

It is important to not only consider laryngeal control in the modification of vocalizations since, for example, human speech is modified by labial and oro-facial control. In this regard, some non-human primates seem to show relatively greater control in modifying the acoustics of their vocalizations and/or to produce non-vocal sounds that do not appear to be innate. Chimpanzees can produce novel attention-getting sounds that are modified by labial (lip) vibrations (Hopkins et al., 2007). This includes a “Raspberry” call where the animals pucker the lips and make a vibrating sound (http://www.youtube.com/watch?v=2Oma_2RFTaM). This call can be imitated by naïve individuals in captivity and some of these calls are also seen in populations in the wild (Marshall et al., 1999; Crockford et al., 2004; Egnor and Hauser, 2004). Consistent with these observations, an orangutan learned to use her lips to copy a novel whistle produced by a human (Wich et al., 2009).

Certainly, relative to song-learning birds, humans and other vocal learners, non-human primates do not fit the stereotyped view of a production vocal learner (Egnor and Hauser, 2004; Snowdon, 2009). Rather, we would interpret the evidence for vocal plasticity and flexibility in some non-human primates as limited-vocal learning, albeit with greater flexibility via non-laryngeal than laryngeal control. But they do not have the considerable levels of laryngeal (mammalian) or syringeal (avian) control as seen in complex vocal learners. We next ask: are there other examples in the animal literature that also do not clearly fit the categorical vocal learning vs. vocal non-learning distinction?

#### Non-primate examples challenging the vocal learning/non-learning distinction

Regarding the so-called vocal *non-learning* birds, there is an interesting report on a suboscine passerine bird with possible evidence of at least limited-vocal learning (Saranathan et al., 2007). Suboscines are the closest relatives of songbirds, like chimpanzees are to humans. Likewise the first suboscine species studied (Eastern Phoebe—Saynoris genus; Kingbird—Tyrannus genus) were found to: (1) not be vocal learners based on social isolation and deafening experiments; and (2) to not have forebrain song nuclei as seen in their close oscine passerine songbird relatives (Nottebohm, 1980; Kroodsma and Konishi, 1991). However, another species belonging to a separate suboscine lineage, the Three-wattled bellbird (Procnias genus) shows conspicuous ontogenetic and geographical song variation and fairly rapid song change within a population, which the authors argue cannot be explained by genetic differences alone (Saranathan et al., 2007). These results suggest that this suboscine species could be a limited- or even moderate-vocal learner, rather than a vocal non-learner. Determining the presence or absence of song nuclei in their forebrain still needs to be investigated.

Mouse ultrasonic vocalizations have recently been described as having “song” or song-like characteristics (Holy and Guo, 2005). However, this does not necessarily mean that mice are vocal learners, because, for example, in birds songs can either be learned or innate (Kroodsma and Konishi, 1991). Kikusui et al. (2011) conducted cross-fostering experiments with mice and did not find evidence of vocal learning (Kikusui et al., 2011). However, recent work by Arriaga and Jarvis (Arriaga and Jarvis, in press; Arriaga et al., in press) on cross-housed males shows that the animals sing their ultrasonic courtship “song” to females with a different pitch in the presence of other males from different strains. For instance, one mouse will match the pitch of his larger male cage mate in the presence of a female. In addition, deafening showed that the mice require auditory feedback to develop and maintain some of the acoustic properties of their song syllables (Arriaga and Jarvis, in press; Arriaga et al., in press). This seems to be limited vocal learning, because the animals appear to be making acoustic changes to innately specified vocalizations.

As another example, a recent report in an ungulate (goats) shows what we believe to be limited-vocal learning (Briefer and McElligott, 2011). The authors studied the social effects of goat vocal ontogeny and note a number of acoustical differences between kids that were placed in different social groups. Goats in the same groups showed more similarity in vocalization acoustics. Here, again changes seem to occur to innate pre-specified vocalization components, to the point that there are considerable differences in the final modified vocalizations relative to the originals.

These examples in the animal behavior literature suggest a greater variability in vocal flexibility than is often appreciated. Certainly, some animals would likely remain in the “vocal non-learner” category, for which there is considerable evidence for a lack of vocal flexibility under different conditions. Yet, findings such as illustrated by the above examples provide support for the vocal learning continuum hypothesis (Arriaga and Jarvis, in press) and we would suggest that certain birds, non-human primates, mice, and goats could be reclassified as either *limited-vocal learners* or *moderate-vocal learners*, including many currently known vocal learners as *complex-vocal learners* (with humans among these as *high-vocal learners*). This hypothesis is illustrated in Figure 1A, where the hierarchically higher the vocal-learning category, the fewer species that are observed in that category. We next consider whether there is any evidence that sensory learning, auditory sequence learning in particular, is similarly distributed across several categories, and if so, how could it have influenced the evolution and mechanisms of vocal learning.

### Complexity in vocal production vs. sensory (auditory) learning: evaluating syntactic-like vocal production and how animals learn artificial grammars

It has been argued that a distinction needs to be drawn between production vocal learning and sensory (e.g., auditory) learning (Jarvis, 2004; Petkov and Wilson, 2012). Auditory learning appears to be more broadly distributed in the animal kingdom than production vocal learning, and, although necessary, it is not sufficient for vocal learning. For instance, certain dogs, in which there is no evidence for complex vocal production learning, can be trained by humans to associate the sounds of spoken names of tens to hundreds of objects and to retrieve either the correctly named objects or novel objects (Kaminski et al., 2004). Almost all animals tested in classical conditioning experiments can learn to make simple sound associations with reward or punishment, such as detecting single sounds or discriminating pairs of differing sounds (Moore, 2004). The question we ask is what is the range of auditory learning complexity across species and how might this relate to human syntactic learning capabilities? In this regard, it is useful to look at the interface of linguistic theory and experiments in evolutionary biology, which aim to address the level of “syntactic-like” sequencing complexity in either vocal production or the sensory learning capabilities of different animals.

Human syntactic abilities allow us to both perceive and produce grammatical relations between words or word parts in a sentence, and linguists distinguish between language competence and language performance (Chomsky, 1965). Modern linguistic theory has been applied to characterize not only human syntactic abilities but also the complexity in vocal production or auditory sequence learning capacities in a variety of non-human animals (Okanoya, 2004; Berwick et al., 2011; Hurford, 2012). For instance, the Formal Language Hierarchy (FLH) contains several categories of grammar (rule-based systems), each describing an increasingly powerful computational language (Chomsky, 1957; Berwick et al., 2011; Hurford, 2012). Lower ranked grammars, called Finite-State Grammars (FSG) are computationally weaker systems that can only generate strings of sequences with limited structural complexity. Higher ranked grammars can also generate the simpler forms of structural complexity but are less limited. Human spoken language is said to encompass the later, as it can have elaborate hierarchical structures with many non-adjacent relationships between sequence elements, such as the nesting of phrases within other phrases (Berwick et al., 2011; Hurford, 2012; Jaeger and Rogers, 2012; Petkov and Wilson, 2012). Such abilities are thought to be unique to humans in both production and perception. Some animal behavioral studies have challenged this perspective, but remain highly controversial (for a review: Berwick et al., 2011; Jaeger and Rogers, 2012; Ten Cate and Okanoya, 2012). We argue that, instead of focusing on the threshold of “human unique” capabilities, further efforts are needed to better resolve the different levels of complexity in the FLH where non-human animal capabilities are likely to vary to a greater extent (see: Hurford, 2012; Jaeger and Rogers, 2012; Petkov and Wilson, 2012). Combined with further comparative testing, this approach could provide novel insights on the relationship between animal sequence learning capabilities either for perception or production and human syntactic capabilities.

#### Structural complexity of animal vocal production

As complex vocal learning groups, songbirds and whales are known to naturally produce sequences of their songs with syntactic-like organization, but the structure of their songs do not seem to be more elaborate than sequences that can be generated by FSGs (or “regular grammars”) (Okanoya, 2004; Bolhuis et al., 2010; Berwick et al., 2011). In other words, unlike humans, non-human animals do not seem to show deeper hierarchical relationships, such as the nesting of song phrases within others. Further, humans can change the meaning of expressions by changing the syntactic organization of the units, called “compositional syntax” (Tallerman, 2011; Hurford, 2012). But the songs of non-human animals have so far been only characterized as “phonological syntax,” since the way that the units are structured are thought not to generate new meanings (Marler, 1970, 2000; Berwick et al., 2011). It remains possible that further experiments with many more species could obtain data to challenge these interpretations of the animal behavioral literature.

As for vocal non-learners or limited-vocal learners, the natural syntactic-like vocal production abilities of non-human primates and many other vertebrates seem to be considerably more limited than those of complex-vocal learners. For example, some species of guenons (Old World monkeys) appear to combine pairs of calls into different context-specific call sequences (Ouattara et al., 2009). Other guenon species use combinations of two alarm calls to elicit group movement in the wild that does not seem to be instigated by the individual calls themselves or by other types of call sequences (Arnold and Zuberbuhler, 2006). Whether other non-human primate species can use and produce combinations of call pairs is currently unknown. It has been suggested that gibbon “song-like” vocalizations contain a different organization of vocalizations when predators are present (Clarke et al., 2006). However, it is not clear whether the information bearing parameters of gibbon songs lie in the proportion of particular song elements and/or the structure of how the elements are organized. Chimpanzees are able to learn to manually combine several learned visual symbols to “sign” with humans (Rumbaugh, 1977), but their ability to do so with vocalizations is considerably more limited and in all cases these abilities require extensive training (Shettleworth, 2010). Therefore, the current impression is that the combinatorial vocal production capabilities of non-human primates are limited to combinations of one to two vocalizations.

#### Artificial-grammar learning and animal sequence learning capabilities

Just as vocal production capabilities seem to vary in complexity across the animal kingdom, auditory and other sensory learning capabilities could considerably vary across species. However, since sensory learning capabilities can be associated with behaviors that are not tied to vocal production, an important question is: how to measure these abilities systematically and in ways that allow cross species comparisons?

Artificial-Grammar Learning (AGL) paradigms (Reber, 1967) are useful for understanding how different individuals learn the structure of a sequence of sensory elements. Artificial Grammars (AG) can be designed to create different levels of structural complexity in how elements are organized in a sequence. The learning of these sequences can be measured using non-vocal motor output (e.g., Fitch and Hauser, 2004; Gentner et al., 2006; Murphy et al., 2008). Generally, these experiments involve an initial phase where the animals are either explicitly trained to learn exemplary “correct” sequences that follow the AG structure, or they are habituated to the exemplary AG sequences. The latter approach aims to tap into more implicit forms of learning, similar to the way that infants glean the statistical properties of language-related structure (Saffran et al., 1996; Marcus et al., 1999). Subsequent to the learning phase, the animals are tested with novel “correct” and “violation” sequences to determine if they can distinguish them, either by their trained or natural responses (e.g., by measuring preferential looking responses towards the different testing sequences). As examples of the types of structures that can be studied with AGL paradigms, AGs can be designed to have only adjacent relationships between the elements in a sequence (Saffran et al., 1999; Fitch and Hauser, 2004; Friederici, 2004; Friederici et al., 2006), non-adjacent relationships between more distantly associated elements (Friederici et al., 2006; Pallier et al., 2011; Petersson et al., 2012), and/or hierarchically organized relationships (Bahlmann et al., 2008, 2009; Friederici, 2011). For further details on the historical basis for and the use of AGL paradigms in adult humans, infants or other animals see: (Reber, 1967; Fitch and Hauser, 2004; Fitch and Friederici, 2012; Petkov and Wilson, 2012).

In a few studies with songbirds, where starlings (Gentner et al., 2006) or Bengalese finches (Abe and Watanabe, 2011) participated in AGL paradigms, it was claimed that these species can learn hierarchically nested grammatical structures. However, these interpretations have been challenged on the grounds that it remains possible that the animals could have learned the difference between “correct” and “violation” sequences by using simpler strategies, which is considered in detail elsewhere (van Heijningen et al., 2009; Berwick et al., 2011; Ten Cate and Okanoya, 2012). Thus, some authors have concluded that it remains controversial whether any non-human animal can recognize auditory patterns that require grammars hierarchically higher than FSGs or regular grammars (e.g., context-free grammars, see Berwick et al., 2011).

Tamarins, a New World monkey species, seem able to perceptually learn adjacent relationships between FSG sequences (e.g., Fitch and Hauser, 2004), although it is not clear if this extends to the learning of non-adjacent relationships (also see: Newport et al., 2004). However, a number of the results on the testing of AGL in non-human primates that have used preferential looking paradigms to measure behavioral responses, have been questioned in part because of the subjective nature of experimenters rating the responses of animals captured on video (Ten Cate and Okanoya, 2012). Wilson and colleagues have devised some solutions to automate the analysis of natural eye-movement responses using non-invasive eye-tracker systems (Wilson et al., 2011). With this approach they have obtained evidence that Rhesus macaques can learn an auditory artificial-grammar with several forward branching relationships, such as those often seen in the produced songs of songbirds and cetaceans (Hurford, 2012). With greater objectivity, it is important to revisit the issue of what level of structural complexity in auditory pattern learning different animals are naturally capable (Petkov and Wilson, 2012).

Regarding what non-human primates are capable of learning with training, an interesting recent report trained baboons on pairwise associations between several visual symbols, e.g., A1-B1, A2-B2, etc. (Rey et al., 2012). In a later testing phase, the animals were presented with the initial “A” elements of two pairs (e.g., A1-A2) and were then allowed to select the “B” elements that would follow. Here, the animals were seen to preferentially pair the “B” partner of the most recent “A” element that was seen (e.g., A2-B2), followed by the partner pair of the first element (e.g., A1-B1). This resulted in the most often selected pattern, A1-A2-B2-B1, which resembles a hierarchical “center-embedded” (or nested) structure. It is interesting that the baboons seemed to rely on an associative memory trace of the pairs of elements that they were trained to recognize, which as the authors interpret may have had an evolutionary basis for human abilities to nest syntactic expressions. However, since FSG are subsets of hierarchically higher grammars and FSGs can generate sequences that can appear to be nested, whether the baboons can learn center-embedded structure remains unclear. Some linguists have outlined a set of criteria on which the animal work would need to be evaluated, if this is the objective (Jaeger and Rogers, 2012). Thereby, as with the related songbird studies (Gentner et al., 2006; Abe and Watanabe, 2011), it is currently unclear whether any non-human animal can learn patterns above those that can be generated by FSGs (or regular languages) in the FLH.

#### A need for continuing revision of the formal language hierarchy combined with further comparative testing

Given that vocal learning and sensory learning capabilities appear to be more variable among vertebrates than is often appreciated (Figure 1), approaches in linguistics and those that rely on AGL paradigms remain useful for clarifying the extent of animal capabilities. However, there are important issues that tend to get overlooked which can limit our understanding of the structure of animal vocalizations or the extent of animal AGL capabilities:

- FSGs are subsets of languages higher on the FLH. Thus it is not always easy to know whether the vocal production or sensory learning of a particular set of sequences requires a higher-level process. Without evidence for a higher-level process a simpler process might be possible both in humans and other animals. For instance, humans can rely on semantics to simplify the complexity of a syntactic process and even humans can find AGL void of semantic content challenging to learn (Perruchet and Rey, 2005; Uddén et al., 2012).
- There has been considerable interest in understanding how high humans and other animals can reach into the FLH. However, by focusing solely on the top end of the FLH, the animal AGL experiments have tended to under-support some of the other potentially interesting aspects in the data on animal AGL. For example, it remains unclear the extent to which non-human animals can learn non-adjacent relationships between sounds, which many view as a key evolutionary transition in the evolution of human syntactic abilities (for a review: Fitch and Friederici, 2012).
- There are considerable levels of structural complexity in FSGs (Reber, 1967; Petersson et al., 2012) that need to be better resolved so that different types of AG structures can be systematically changed and/or compared to others (Hurford, 2012; Petkov and Wilson, 2012).

Some groups have been considering how the FLH can be resolved in greater detail (see: Hurford, 2012; Jaeger and Rogers, 2012; Petkov and Wilson, 2012). For example, Petkov and Wilson (2012) note that the simplest scenario for auditory learning is the recognition of a single sound/element, such as the recognition of a single vocalization from a limited set of vocalizations. With the recognition of two types of elements in a sequence, it is known that many animals habituate to the repetition of the same element and dishabituate to the introduction of a novel element (e.g., repetition effects, Grill-Spector et al., 2006). With three or more different elements, there is the possibility of creating a greater number of structural relationships in the transitions between elements. Continuing efforts are needed to quantify the multidimensional space of “syntactic complexity,” especially for FSG structures where animal abilities vary. A better understanding of the graded levels of “syntactic complexity” in vocal production and sensory learning capabilities across species could clarify the origins of syntax and spoken language.

### Evolutionary hypotheses on vocal and auditory learning: gains, losses or everyone has it?

**Figure 2:**
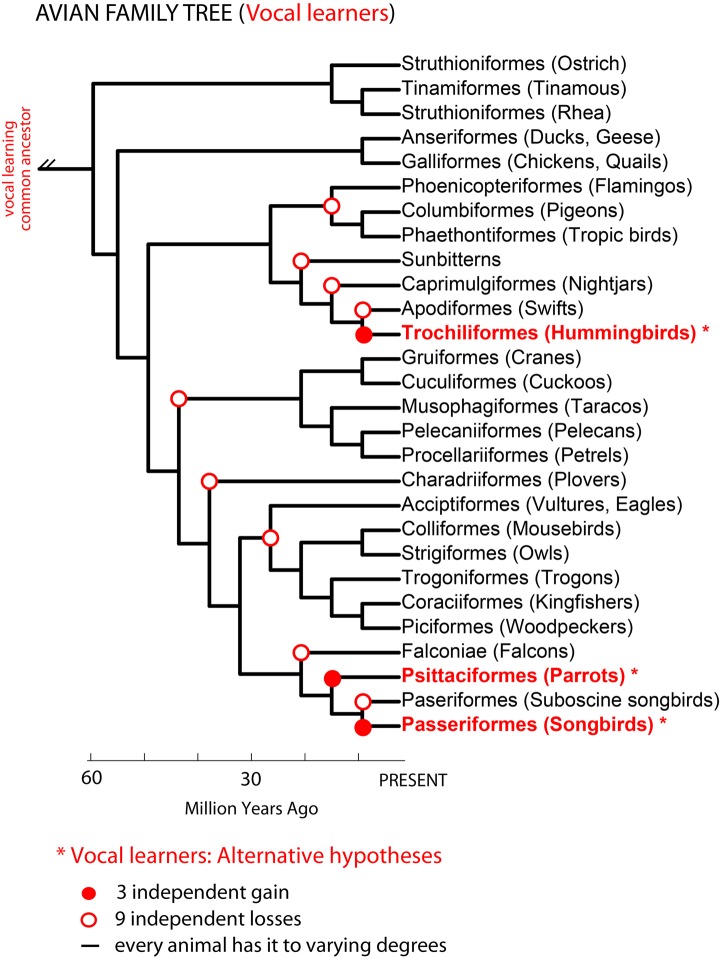
**Avian phylogenetic tree and the complex-vocal learning phenotype.** Shown is an avian phylogenetic tree (based on: Hackett et al., 2008). Identified in red text and ^*^ are three groups of complex-vocal learning birds. Below the figure are summarized three alternative hypotheses on the evolutionary mechanisms of complex-vocal learning in birds (see text, and Jarvis, 2004). The auditory sequence learning phenotype described in Figure 1B, is not shown here, since some forms of auditory learning seem to be present in all birds. However, further comparative data is needed on the learning of the complexity of auditory sequences, which to our knowledge has been tested using Artificial Grammars only in songbirds (Gentner et al., 2006; van Heijningen et al., 2009; Abe and Watanabe, 2011).

Phylogenetic comparisons suggested that complex-vocal learning evolved among birds at least two, if not three independent times: in oscine songbirds, parrots, and hummingbirds (Nottebohm, 1976; Jarvis et al., 2000; Hackett et al., 2008; Suh et al., 2011). The difference in the number of independent vocal learning events depends on the interpretation of different phylogenetic trees (Figure 2): (1) either three gains in all three lineages based on phylogenetic trees that are separated by multiple non-learners (Sibley and Ahlquist, 1990; Jarvis, 2004) or (2) two gains, in hummingbirds and the common ancestor of parrots and oscine songbirds, with a loss in the suboscine songbirds (Suh et al., 2011). To explain either of these observations, Jarvis (2004) proposed at least three not mutually exclusive hypotheses for the evolution of vocal learning: (1) complex vocal learning independently evolved multiple times in birds; (2) complex vocal learning was lost either four (Jarvis, 2004) or nine times (Suh et al., 2011); and/or (3) all species are vocal learners to some extent. We note that vocal learning being independently gained or lost suggests a categorical distinction between vocal learners and vocal non-learners. Vocal learning being more continuously distributed among many species than categorical would indicate that gains and losses can occur to a greater extent.

**Figure 3:**
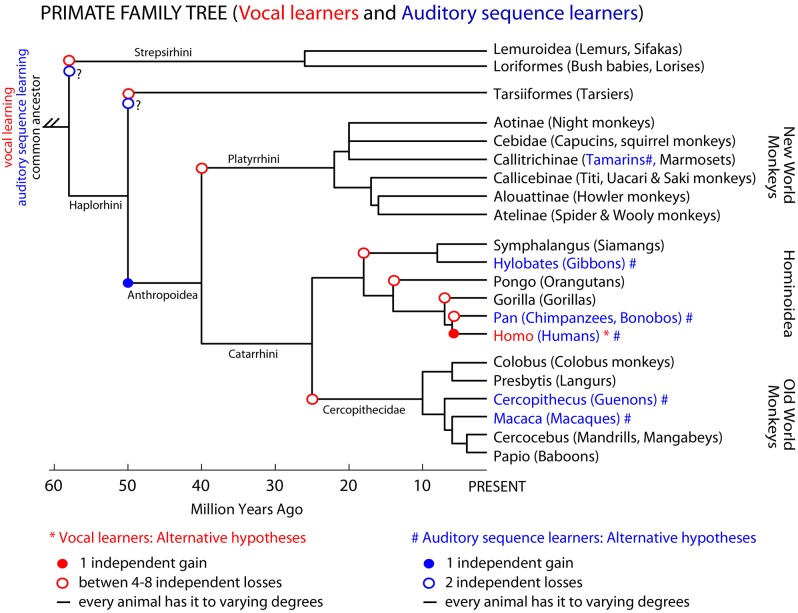
**Primate phylogenetic tree and complex-vocal learning vs. auditory sequence learning.** Shown is a primate phylogenetic tree based on a combination of DNA sequence and fossil age data (Goodman et al., 1998; Page et al., 1999); for a recent review see (Cartmill, 2010). Humans (Homo) are the only primates classified as “vocal learners.” However, non-human primates might be better at auditory sequence learning than their limited vocal-production learning capabilities would suggest. In blue text and (#) we highlight species for which there is some evidence of Artificial Grammar Learning capabilities for at least adjacent relationships between the elements in a sequence (tamarins: Fitch and Hauser, 2004), (macaques: Wilson et al., 2011). Presuming that the auditory capabilities of guenons and gibbons mentioned in the text (or the symbolic learning of signs by apes) would mean that these animals are able to learn at least adjacent relationships in Artificial Grammars we can tentatively mark these species also in blue #. Note however, that for the species labeled in black text, future studies might show them to be capable of some limited-vocal learning or various levels of complexity in learning the structure of auditory sequences. Three not mutually exclusive hypotheses are illustrated for both complex-vocal learning and auditory sequence learning.

**Figure 4:**
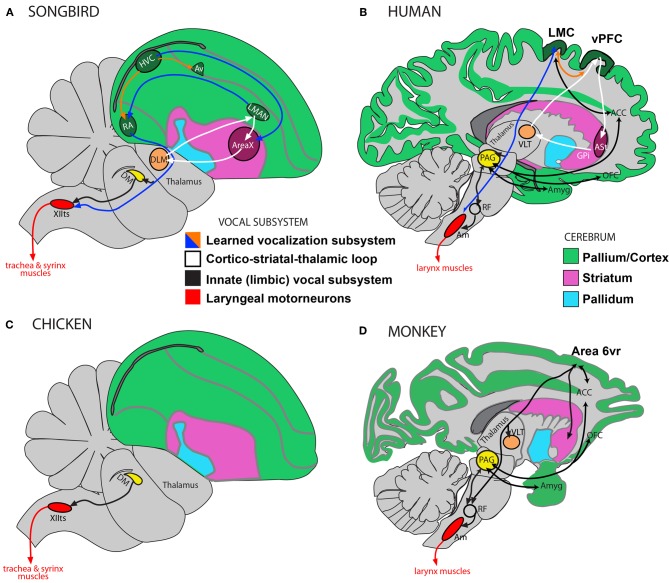
**Vocalization subsystems in complex-vocal learners and in limited-vocal learners or vocal non-learners: Direct and indirect pathways.** The different subsystems for vocalization and their interconnectivity are illustrated using different colors. **(A)** Schematic of a songbird brain showing some connectivity of the four major song nuclei (HVC, RA, AreaX, and LMAN). **(B)** Human brain schematic showing the different proposed vocal subsystems. The learned vocalization subsystem consists of a primary motor cortex pathway (blue arrow) and a cortico-striatal-thalamic loop for learning vocalizations (white). Also shown is the limbic vocal subsystem that is broadly conserved in primates for producing innate vocalizations (black), and the motoneurons that control laryngeal muscles (red). **(C)** Known connectivity of a brainstem vocal system (not all connections shown) showing absence of forebrain song nuclei in vocal non-learning birds. **(D)** Known connectivity of limited-vocal learning monkeys (based on data in squirrel monkeys and macaques) showing presence of forebrain regions for innate vocalization (ACC, OFC, and amygdala) and also of a ventral premotor area (Area 6vr) of currently poorly understood function that is indirectly connected to nucleus ambiguous (see text). The LMC in humans is directly connected with motoneurons in the nucleus ambiguus, which orchestrate the production of learned vocalizations (also see Figure 5B). Only the direct pathway through the mammalian basal ganglia (ASt, anterior striatum; GPi, globus palidus, internal) is shown as this is the one most similar to AreaX connectivity in songbirds. Modified figure based on (Jarvis, 2004; Jarvis et al., 2005). Abbreviations: ACC, anterior cingulate cortex; Am, nucleus ambiguus; Amyg, amygdala; AT, anterior thalamus; Av, nucleus avalanche; DLM, dorsolateral nucleus of the medial thalamus; DM, dorsal medial nucleus of the midbrain; HVC, high vocal center; LMAN, lateral magnocellular nucleus of the anterior nidopallium; LMC, laryngeal motor cortex; OFC, orbito-frontal cortex; PAG, periaqueductal gray; RA, robust nucleus of the of arcopallium; RF, reticular formation; vPFC, ventral prefrontal cortex; VLT, ventro-lateral division of thalamus; XIIts, bird twelfth nerve nucleus.

In the primate phylogenetic tree, only humans are thought to be complex-vocal learners (Figure 3 solid red circle). As with birds, one possible evolutionary hypothesis is that humans evolved vocal learning independently from other primates. Alternatively, if we suppose that a primate ancestor was a complex vocal learner, complex-vocal learning would have to have been lost at least eight times in the primate order (Figure 3, open red circles) and maintained in humans. The evolutionary losses hypotheses become less tenable when the number of losses greatly exceeds the number of independent gains. Putting this together, according to these phylogenies and vocal phenotypes, the number of independent gains is: 1 in primates (Figure 3), 2–3 in birds (Feenders et al., 2008), and 5 in mammals including humans (Jarvis, 2004). The number of losses can be as high as: 8 in primates (Figure 3), 4 or 9 in birds (Feenders et al., 2008; Hackett et al., 2008), and 11 in mammals (Fitch and Jarvis, in press). If the losses are true, what could explain such high rates of losses? One idea is that predatory influences may have selected against vocal learning by selecting against complex vocalization sequences that would allow predators to better localize their prey (Hosino and Okanoya, 2000; Jarvis, 2004, 2006). Some support for this notion is that the known mammalian vocal learners (humans, elephants, and cetaceans) are at or near the top of the food chain, and some of the avian vocal learners (corvid songbirds, hummingbirds, and parrots) are considered exceptional at escaping predators (Jarvis, 2006). Nonetheless, the evolutionary mechanisms may not necessarily be the same across animal species.

Similar forms of gains, losses or other hypotheses could be applied to auditory (sequence) learning abilities. However, here there is a greater paucity of comparative data. Non-human animals may considerably differ in their ability to learn the various levels of sequencing complexity in AG, which at face value could be considered to have evolved independently or by common descent (Figure 3). In several non-human primate species there is an impression of at least the ability to learn adjacent auditory relationships in AG structures (Figure 3, blue nodes and text). Some of these species have also been shown to have relatively simple combinatorial production capabilities (Arnold and Zuberbuhler, 2006). However, we are not aware of evidence for or against prosimians (lemurs, bush-babies, etc.) being able to perceptually learn various levels of structural complexity in AGs or to produce simple sequences with their vocalizations. Thus, additional comparative study is needed to fill in this currently tentative picture (Figure 3). In this regard, as we have argued, developments in linguistic theory and AGL approaches can help us to characterize the extent of the syntactic-like capabilities of non-human animals either for production or sensory learning. We further argue that understanding the distinctions in such behavioral phenotypes and their mechanisms across species will require an improved understanding of their neurobiological substrates.

## Neurobiological pathways for vocal production

**Figure 5:**
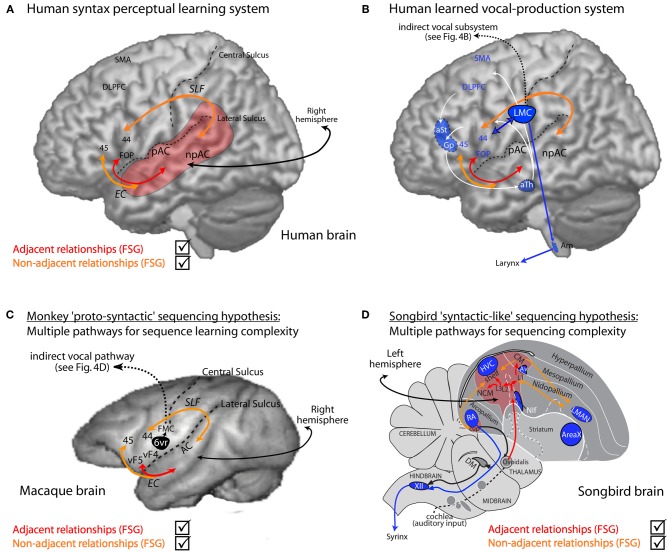
**Human syntactic learning and vocal production sub-systems, with hypothesized monkey and bird evolutionary substrates. (A)** Auditory perceptual learning system in humans (red and orange). Primary (pAC) and non-primary (npAC) auditory cortical regions are engaged in the auditory perceptual organization of sound. **(B)** The perceptual learning system interacts with a system for learned vocal production (blue, also see Figure 4B). **(C)** Hypothetical evolutionary “proto-syntactic” pathways that might be engaged in monkeys for the perceptual learning of different auditory sequence structures in Finite-State Artificial-Grammars (FSG), e.g., adjacent (red text) vs. non-adjacent (orange text) relationships (also see text). Note that the hypothetical ventral pathway is not expected to directly engage monkey Area 6vr (black) or the innate vocal production subsystem (black; see Figure 4D). More bilateral hemispheric engagement might be expected in non-human primates, see text, and/or that the cortical-striatal-thalamic loop would also be engaged in certain forms of implicit sequence learning. **(D)** Songbird auditory (red region and red/orange arrows) and song motor (blue regions) pathways. The auditory pathway is proposed to interact with motor regions adjacent to song nuclei for syntactic-like processing and production of vocal or non-vocal behaviors. Abbreviations: AC, auditory cortex; EC, extreme capsule fasciculus; SLF, superior-longitudinal fasciculus; UF, uncinate fasciculus. CM, caudal mesopallium; DLPFC, dorso-lateral prefrontal cortex; FMC, face motor cortex; FOP, frontal operculum; L2/L3, fields L2 and L3; NIf, interfacial nucleus of the nidopallium; NCM, caudal medial nidopallium; SMA, supplementary motor area; vF4/vF4, macaque anatomical regions ventral F4/F5; 44, 45, Brodmann Areas; See Figure 4 for further abbreviations.

Humans heavily rely on a forebrain pathway to produce learned vocalizations. This pathway is thought to be in many ways separate from an ancestral pathway in non-human primates for producing innate vocalizations (Jurgens, 2002; Jarvis, 2004). Similarly, complex-vocal learners such as songbirds, parrots, and hummingbirds have distinct vocal learning forebrain nuclei that have so far not been found in other birds. That is, for birds, despite the noted variability in the behavioral evidence for vocal learning (Figure 1), the published neurobiological evidence has highlighted distinctions between the neurobiological substrates for vocal production in so-called vocal learners and vocal non-learners (Figures 4, 5A,B). We overview this literature here, which might be challenged or supported by future work.

### Different subsystems and direct and indirect pathways for vocalization in primates

Historically, our understanding of the brain pathways involved in the production of innate vocalizations in primates stems from the classical brain stimulation studies of Penfield and colleagues (Penfield and Rasmussen, 1949) and the anatomical studies of Kuypers in human and non-human primates (Kuypers, 1958a,b, 1982). These studies were followed by over 40 years of work by several groups in different monkeys (such as squirrel monkeys and macaques) using anatomical tracing, neurophysiological recordings during vocalization, lesions to affect vocalizations, and microstimulation to either elicit vocalization or to contract laryngeal muscles (for reviews: Jurgens, 2002, 2009; Simonyan and Horwitz, 2011). Others compared the results in primates (Hast et al., 1974) with those from cats and dogs (Milojevic and Hast, 1964), suggesting some key differences between the human, monkey, and carnivore vocal production subsystems.

Primates are thought to have a limbic (affective) or innate vocal-production subsystem (Figures 4B,D) that involves connections from the amygdala, orbito-frontal cortex and anterior-cingulate cortex to the periaqueductal gray (PAG) in the brainstem (Jurgens and Pratt, 1979b,a; Kirzinger and Jurgens, 1982, 1985). Neurons in the PAG synapse onto neurons in the reticular formation, which in turn synapse with the α-motoneurons in the nucleus ambiguus (Dujardin and Jurgens, 2005). The motoneurons in the nucleus ambiguus control the muscles of the larynx for vocal production (Figures 4B,D). The PAG and reticular formation are required for the motor production of vocalizations via nucleus ambiguus (Jurgens, 2002; Hage and Jurgens, 2006; Hannig and Jurgens, 2006).

Non-human primates also have a ventro-rostral cortical region in Brodmann Area 6 (Area 6vr) that projects to the vocal production areas of the reticular formation, which in turn projects to nucleus ambiguus (Simonyan and Jurgens, 2003), see Figure 4D. When this area in non-human primates is stimulated, it contracts the laryngeal muscles (Hast et al., 1974). Area 6vr and the anterior cingulate are also interconnected with parts of the primary motor cortex, amygdala, and ventral and midline thalamus. However, when Area 6vr is stimulated no vocalizations are produced (Hast et al., 1974; Simonyan and Jurgens, 2003), and when it is lesioned vocalizations are reportedly unaffected (Jurgens, 2002). Thus, it has been proposed that Area 6vr controls respiration associated with vocalization rather than the control of vocalization (Jurgens, 2002, 2009).

However, there is growing evidence that, in non-human primates, neurons in Area 6vr or surrounding regions in the ventral prefrontal/premotor cortex of non-human primates can be modulated during innate vocalization production; although at this point it is not clear whether the results depend on the animals hearing their own vocalizations or the context surrounding the vocalizations. A recent study in macaques suggests that when monkeys produce innate vocalizations on cue, some neurons show premotor neural activity in a frontal cortical area near or in Area 6vr (Coude et al., 2011). Notably, the animals made the vocalizations during a learned non-vocal task and the activity response only occurred under certain conditions. Two other studies in common marmoset monkeys (*Callithrix jacchus*) reported on neuronal activity-related gene expression patterns during vocalization. In one of these studies higher numbers of *egr*-1 immunopositive cells were observed in the prefrontal cortex when the animals vocalized relative to when they remained silent (Simões et al., 2010). In the other study, *c-fos* induction was higher in the ventrolateral prefrontal cortex during vocal perception and production (“anti-phonal” calling) than during vocal production alone, which had higher *c-fos* induction in dorsal premotor cortex (Miller et al., 2010). These findings suggest that the sensory input into these regions may be an important factor for neuronal activation. It has thus become important to determine whether sensory input or motor activity during vocalization is primarily responsible for the observed results by temporarily deafening the vocalizing animals with ear plugs or some other ethically acceptable manipulation. Such future work is likely to clarify the functional role of the prefrontal/premotor cortex, including Area 6vr, during vocalization in non-human primates.

Humans are thought to also rely on the innate vocal-production pathway (e.g., cingulate, amygdala, PAG, and nucleus ambiguus) for producing involuntary vocalizations, such as, when a person shrieks to an aversive stimulus. However, humans rely considerably more on another pathway for learned vocalization, i.e., speech production. This pathway includes the primary motor cortex, regions in the lateral inferior and middle frontal cortex, premotor cortex, supplementary motor cortex, cerebellum, and subcortical structures such as, parts of the basal ganglia and thalamus (Jurgens, 2002; Jarvis, 2004; Simonyan and Horwitz, 2011). In humans, this so-called learned vocal pathway appears to have a direct projection from the face area of primary motor cortex in Brodmann Area 4 to the nucleus ambiguus (Kuypers, 1958a; Iwatsubo et al., 1990; Jurgens, 2002; Khedr and Aref, 2002). This human region in BA4 is also called the Laryngeal Motor Cortex (LMC; Figure 4B). When it is stimulated the participants vocalize (reviewed in: Simonyan and Horwitz, 2011). When human LMC has been damaged the production of learned vocalizations is eliminated. No such homolog of the LMC region has been found in the primary motor cortex of non-human primates, either with stimulation or by lesion. Homologs of human LMC or the non-human primate Area 6vr have been searched for in some non-primate mammalian species, such as cats and dogs, but have not been found (Milojevic and Hast, 1964).

Based on these findings, many investigators hypothesized that the evolution of spoken language in humans was associated with the formation of a direct projection from LMC to nucleus ambiguus in humans (Kuypers, 1958a; Kirzinger and Jurgens, 1982; Iwatsubo et al., 1990; Jurgens, 2002; Khedr and Aref, 2002; Jarvis, 2004; Okanoya, 2004; Fitch et al., 2010; Fischer and Hammerschmidt, 2011; Simonyan and Horwitz, 2011). Simonyan and Horwitz (2011) (also see: Simonyan and Jurgens, 2003), hypothesized that the *function* of the Area 6vr region migrated from its presumed ancestral premotor cortex location in non-human primates into the primary motor cortex to become the LMC in humans, simultaneously taking over direct control of the nucleus ambiguus. To test this hypothesis, further work is needed to clarify whether humans have an area with the functionality and connectivity of non-human primate Area 6vr for contracting the laryngeal muscles. Also, the functional significance of the direct projection in humans from LMC to the nucleus ambiguus remains unclear, relative to the indirect projection from Area 6vr in other primates. Direct motor cortex control of motorneurons controlling hand and finger movement is seen to various extents in both human and non-human primates but less so in rodents (Lemon, 2008). However, a recent finding in laboratory mice appears to have revealed an LMC-like region (Arriaga et al., in press), which is active by vocalization production and makes a direct, but very sparse, projection to nucleus ambiguus, also see: (Arriaga and Jarvis, in press). This finding motivates a re-evaluation of the origins of the LMC in humans. In this regard, although a number of studies state that in non-human primates there is an absence of a direct projection from motor cortex to nucleus ambiguus, Kuypers' original 1958b study mentioned finding some peri-central cortical axons in the nucleus ambiguus of monkeys and chimpanzees. These findings can support the continuum hypothesis of vocal learning: Arriaga and Jarvis (in press) hypothesize that in addition to the presence vs. absence of the direct nucleus ambiguus projection, the density of the projection would be correlated with the level of limited to more complex vocal learning.

### Distinct Vocal Forebrain Nuclei in complex-vocal learning birds

The differences in neural pathway connectivity for complex-vocal learning and limited-vocal learning or vocal non-learning birds are seen to parallel some of the findings in mammals. Best studied in songbirds and parrots, the neurobiological substrates for vocal control and learning includes four nuclei in an anterior forebrain pathway loop (which interconnect the pallium with the basal ganglia and thalamus) and three in a posterior pathway of which the robust nucleus of the arcopallium (RA) makes a direct projection onto the vocal motoneurons in the twelfth nerve nucleus (XIIts) of the bird brainstem, which control the muscles of the syrinx (Figure 4A, also: Jarvis, 2004; Bolhuis et al., 2010). No such nuclei or direct projections have been found in so-called vocal non-learning birds, such as ducks and pigeons (Wild, 1997; Dubbeldam, 1998; Jarvis, 2004). This direct projection is reminiscent of the direct projection in humans from LMC to the nucleus ambiguus that appears to be absent in non-human primates. All birds studied to date, however, have been shown to have brainstem input from the midbrain region DM (dorsal medial nucleus of the midbrain) to XIIts (Figures 4A,C), which, like the mammalian PAG projection to the nucleus ambiguus (via the reticular formation), controls the production of innate vocalizations. These cross species differences have been used to strengthen the hypothesis on the evolution of the direct projection being crucial for the evolution of vocal learning (Wild, 1997; Jarvis, 2004; Fitch et al., 2010).

To gain insights into the evolutionary bases of the vocal learning nuclei, Feenders and colleagues (2008) compared the forebrain vocal nuclei and adjacent brain regions in animals from each complex-vocal learner lineage—songbirds, parrots, and hummingbirds—to so-called vocal non-learners such as doves or non-singing female songbirds.^1^ Extending prior studies (Jarvis and Nottebohm, 1997; Jarvis and Mello, 2000; Jarvis et al., 2000) the authors made the following key observations: First, when vocal learning birds performed non-vocal movement behaviors, such as hopping and flying, expression of the *egr*1 immediate early gene (associated with increases in neuronal activity) was restricted to forebrain regions surrounding or directly adjacent to the forebrain song learning nuclei. Second, in the vocal non-learning birds, comparable activated regions in non-vocal movement areas were found, but without the presence of forebrain song nuclei adjacent to them. Third, the activity-dependent gene activation in these regions was motor-driven and was independent of at least auditory or visual input. The *egr*1 expression in the movement-activated regions was correlated with the amount of body movements (e.g., wing beats) performed, whereas in the song-learning nuclei it was correlated with the amount of singing performed. Lastly, both the vocal learners and non-learners were found to have forebrain auditory pathways that are activated when the animals heard vocalizations, and with no noted differences between vocal learners and non-learners.

Feenders and colleagues (2008) used these results to propose a motor-theory of vocal learning origin. They propose that the brain regions in the vocal learning pathway derived from the same cell lineages that gave rise to the motor pathway for movement control unrelated to vocal production in birds. They propose that the new pathway then formed a direct projection onto the brainstem vocal motor neurons for greater control of vocal production. However, the equivalent function of the non-human primate Area 6vr (which when stimulated contracts laryngeal muscles) and its indirect projection to the vocal motoneurons, has yet to be found in so-called vocal non-learning birds (compare Figure 4C in chickens to 4D in monkeys).

Other factors have been proposed to differ between vocal learners and non-learners, a common factor being hemispheric lateralization. It is known that in both humans and song learning birds there is a dominant hemisphere for learning, production, and processing of vocalizations, being left dominant in humans and canaries, and right dominant in zebra finches (Nottebohm et al., 1976; Simpson and Vicario, 1990; Phan and Vicario, 2010). Some have suggested that the stronger engagement of the left hemisphere in human language processing was a recent evolutionary adaptation (Tyler et al., 2011). This predicts a more bilateral engagement in the brains of limited-vocal learners that are closely evolutionarily related to humans. However, although lateralized functions for non-vocal behaviors have been seen in many species (Halpern et al., 2005), lateralized processing of communication signals in non-human primates, for instance, is sometimes (Heffner and Heffner, 1984; Poremba et al., 2004; Joly et al., 2012) but not always seen or explicitly tested for (for a review see: Petkov et al., 2009). Although lateralization is not restricted to humans, or to vocal learners, the question that remains is whether the level of lateralization, rather than the particular hemisphere, might be the critical variable for differences between complex-vocal learning and other species (Teufel et al., 2010). The ability to simultaneously image both hemispheres in birds, primates and other animals (e.g., Petkov et al., 2006; Boumans et al., 2008; Poirier et al., 2009; Baumann et al., 2011) can provide data for testing hemispheric effects.

### Summary of vocal production pathways in birds and primates

We saw in sections “Different Subsystems and Direct and Indirect Pathways for Vocalization in Primates” and “Distinct Vocal Forebrain Nuclei in Complex-Vocal Learning Birds” that primates and birds appear to share a broadly conserved pathway for producing innate, emotionally or spontaneously driven vocalizations. However, humans and song-learning birds appear to rely considerably more on a forebrain motor system for learned vocalization. The learned vocal-production subsystem has different connectivity with the motor neurons of the laryngeal (in mammals) or syringeal (in birds) muscles than the innate vocal-production subsystem. In all birds, the adjacent forebrain pathway appears to orchestrate motor action unrelated to vocal production, such as, wing flapping or hopping, both of which require movement coordination (Feenders et al., 2008). The same might be the case for primates although this is currently unknown. Various authors (Farries, 2004; Jarvis, 2004; Feenders et al., 2008) have suggested that the simplest evolutionary mechanism for vocal learning is that a genetic mutation established the link between the newly evolved forebrain nuclei and the vocal motor brainstem nucleus for vocal production (compare Figure 4A in songbirds to 4C in chickens). In sections “Summary of Motor and Other Theories” and “Predictions of Motor and Other Theories, From a Modified Behavioral Perspective” we consider this and other, not mutually exclusive, hypotheses, which is based on re-evaluation of motor and cognitive theories that make different predictions about the neurobiological systems for production and perceptual learning.

### Auditory input into the vocal production pathways

Because auditory learning is necessary but not sufficient for vocal learning, one might expect the auditory pathways to provide input into the vocal learning system in the complex-vocal learners but perhaps not for animals that are obligate vocal non-learners. Such auditory input has been the topic of extensive investigation in songbirds and parrots, but without yet a clear resolution (Jarvis, 2004; Mooney, 2009; Margoliash and Schmidt, 2010). In songbirds, the forebrain auditory pathway provides input into the interfacial nucleus of the nidopallium (NIf; a song nucleus) including the high vocal center (HVC) shelf and RA cup regions adjacent to the vocal motor pathway nuclei HVC and RA (Figures 4, 5). The shelf and cup in turn are thought to send weak projections into HVC and RA, whereas NIf sends a strong projection into HVC (Vates et al., 1996; Jarvis, 2004; Mooney, 2009; Yip et al., 2012). Relatedly, human neuroimaging studies have described auditory cortex input into the frontal speech production areas (e.g., Rauschecker and Scott, 2009; Friederici, 2011; Tyler et al., 2011).

In so called vocal non-learners or limited vocal learners, there is considerable data on the structure and function of the auditory pathway from cochlea to cortex, including in non-human primates and other vertebrates (e.g., Rauschecker, 1998; Carr and Code, 2000; Kaas and Hackett, 2000) and song learning birds and pigeons (Mooney, 2009; Margoliash and Schmidt, 2010). In all of these sets of species, the auditory pathway projects from the cochlea to the midbrain auditory nucleus, to the thalamic auditory nuclei, and then to primary and secondary auditory cortical/pallial regions. After entering the forebrain, in vocal non-learners auditory input is thought to enter motor pathways, but in the complex-vocal learners it also enters the vocal motor pathways. If the presumed vocal non-learners are thought to primarily rely on an innate vocal-production system, then auditory input into the vocal production system would not seem to be required for genetically regulated vocal production.

To clarify the neurobiological substrates for auditory processes, vocal production learning, and the interface of the two, it has become critical to: (1) determine which animals are strictly vocal non-learners; (2) whether the neurobiological vocal production pathways in complex-vocal learners are as clearly distinct from those of limited-vocal learners as they seem; and (3) if there are differences across the species in the dependence of the vocal production subsystems on input from the auditory system. These clarifications are needed because the distinctions between “vocal learners” and “vocal non-learners” in their sensory-motor (e.g., auditory-vocalization) interactions are at the core of certain motor, gestural and cognitive theories.

## Summary of motor and other theories

Motor theories are appealing for explaining sensory-motor relationships in communication for the following reasons. The sequencing of motor behaviors at multiple scales is an ancestral function. For example, many quadruped mammals increase their speed of movement by shifting from a walking gait to a running gait, each requiring different coordinated sub-movements of the limbs and sensory-motor feedback (Schmitt, 2010). Human language involves the temporal sequencing of laryngeal and other oral-facial muscles, and respiratory apparati, to produce speech sounds at multiple temporal sequencing levels, including phonological, sub-lexical and lexical, and syntactic. These forms of sequencing are used for perception and production. In the case of language syntax perception, humans often evaluate hierarchically organized dependencies between words in a sentence that cannot be simply solved by sequentially evaluating the words (Bickerton, 2009). Language production also requires coordinating a series of muscle movements of the larynx with feedback from the sensory system. Thereby spoken-language perception and production depend on sensory-motor interactions and these are differently emphasized by the various theories.

Although there are several motor theories in the literature, in this section we compare two sets of not mutually exclusive theories: motor theories of speech/song perception (Liberman and Mattingly, 1985; Williams and Nottebohm, 1985), and a motor theory of vocal learning origin (Feenders et al., 2008). As variants of motor theories, we briefly overview the “gestural theory of spoken language evolution” (Hewes, 1973) and the “gestural (mirror neuron) hypothesis of language evolution” (Rizzolatti and Arbib, 1998). Then we compare them with alternatives to motor/gestural theories, namely broadly conserved “sensorimotor integration” and “cognitive domain general” hypotheses.

### Motor theories of speech/song perception

The well-known motor theories of speech perception in humans (Liberman and Mattingly, 1985) and song perception in songbirds (Williams and Nottebohm, 1985), make the strong claims that speech and song perception are primarily driven by the motor system. Although, one might expect the perception of speech sounds to be a perceptual problem for the auditory system, Lieberman and Mattingly argue that it is difficult to explain a large set of speech perception phenomena by only their sensory representation, since speech perception more often departs from its sensory representation than does the perception of other sounds. The theory proposes that the sensory-motor transformations made during speech perception and production are overlearned in humans. Because of this, the motor system actually drives auditory representation of speech to expedite the perception of speech in a way that is not available for the perception of other sounds.

Others have aimed to generalize the motor theory for speech perception to syntax perception. Allott suggested that the motor system would be important for the perceptual sequencing of syntactic expressions and for preparing syntactically organized sentences for production (Allott, 1992). An interesting variant of the motor theory of speech perception argues that the motor cortex is not necessary for speech perception, *per se*, but is necessary to sequence a conversation between two speakers, such as controlling when the speakers take turns in a conversation (Scott et al., 2009). The motor theory of song perception in songbirds as originally proposed was based on observations that the entire song learning system (from HVC to the descending pathway involving the vocal motoneurons in nucleus XIIts) shows song selective auditory responses (Williams and Nottebohm, 1985); for reviews see Mooney, 2009; Margoliash and Schmidt, 2010.

### Motor theory of vocal learning origin across species

Similar to the motor theory proposed for vocal learning origin in birds (section “Distinct Vocal Forebrain Nuclei in Complex-Vocal Learning Birds”), the same authors proposed a similar theory for humans based on consideration of the evidence in the human literature (Feenders et al., 2008). Like in birds, the theory proposes that humans rely on a speech/song-learning pathway that is based on elaboration of a pre-existing motor pathway that controls learned movement sequencing. This would mean that vocal non-learning birds and mammals only have the forebrain motor pathway that supports movement patterning abilities unrelated to those for vocal production. By comparison, vocal learners evolved a new pathway in parallel to control the vocal motor neurons. In essence, in this theory, like mechanisms of gene evolution, the vocal learning pathway in birds and humans is seen as forebrain motor pathway duplication that adapted to directly control the muscles of the larynx/syrinx in addition to other muscle groups for respiration.

### Gestural theories

There are at least two independently developed gestural theories of language evolution: (1) The general “gestural theory of spoken-language origin” (Hewes, 1973; Tomasello et al., 1993); and (2) The gestural mirror neuron hypothesis of language evolution (Arbib, 2005; Prather et al., 2008; Arbib, 2010). The general gestural theory proposes that the brain pathways controlling the production of speech emerged from ancestral brain pathways controlling learned gestures. Thereby human and some non-human primates can perform learned gestures, but only humans can learn vocalizations relying on the gestural motor system. This theory is similar to the motor theory of vocal learning origin (Feenders et al., 2008). However, the two theories differ in that the gestural theory implies that the brain regions supporting gesturing and speech perception overlap, whereas, the motor theory implies that the more general movement control system was adapted for spoken language.

The gestural mirror neuron hypothesis tries to explain the evolutionary mechanisms of speech production learning by relying on “mirror neuron” results in primate and, more recently, avian vocal motor imitation (Arbib, 2005; Prather et al., 2008; Arbib, 2010). This theory was developed from the discovery in non-human primates that the same neurons fire both when the same action is observed or produced (di Pellegrino et al., 1992). Such neurons have been observed in frontal and parietal cortex. In humans, brain imaging has been used to localize regions presumably containing mirror neurons (e.g., Chong et al., 2008). The gestural mirror neuron hypothesis of language evolution argues that brain pathways that generate speech also process speech and visual gestures, and by doing so are able to transfer and copy hearing or seeing into motor behavior in the motor pathways. Non-human primates are said to have this system, but only for visual to motor copying of neural signals. Humans are said to have it for both visual and auditory to motor copying of signals, including for spoken language (Rizzolatti and Arbib, 1998; Arbib, 2005). The lack of the auditory-vocal motor link in non-human primates is thought to result from a lack of, or weakness of, a link in the auditory to vocal motor pathway, rather than the absence of a vocal motor pathway. In songbirds, Prather and colleagues have found a direct vocal motor link in mirror-neurons. They discovered neurons in the song nucleus HVC that have comparable responses to the production of learned songs and to hearing the songs (Prather et al., 2008). However, the relationship between auditory to vocal mirror neurons in the vocal motor pathway in primates remains largely theoretical (Rizzolatti and Arbib, 1998; Arbib, 2005, 2010).

### Sensorimotor integration and cognitive “domain-general” hypotheses

The above motor and gestural theories differ in the mechanisms of sensory-motor interactions, but share the notion that the auditory-motor interactions in humans and other vocal learning animals have specialized considerably relative to those in other animals. Rauschecker and Scott have proposed a “sensorimotor integration” model (Rauschecker and Scott, 2009; Rauschecker, 2011) that highlights the broadly conserved aspects of auditory-motor processing in human and non-human primates. This model builds on the notion of evolutionarily conserved auditory pathways in human and non-human primates (Romanski et al., 1999; Tian et al., 2001; Rauschecker and Scott, 2009) and other mammals (e.g., cats, Lomber and Malhotra, 2008). They propose that a ventral auditory pathway from the temporal lobe to ventral prefrontal cortex is engaged in processing auditory “objects” (such as calls in animals and speech in humans) and a dorsal auditory-to-premotor pathway for auditory-to-motor interactions that includes language-related processing in humans (for reviews see: DeWitt and Rauschecker, 2012; Rauschecker, 2012). The “sensorimotor integration” model proposes that the dorsal pathway receives efference copies from prefrontal and premotor regions that can affect auditory processing and perception. Such models emphasize the commonalities across the species regarding sensorimotor integration (Rauschecker and Scott, 2009; Rauschecker, 2011). An analogous, but also different efference copy model has been proposed for songbirds. Instead of an efference copy being sent back to the auditory pathway, the copy is thought to be sent from the song nucleus HVC to the anterior forebrain pathway through the basal ganglia for comparing planned motor output with auditory feedback (Troyer and Doupe, 2000; Fee, 2012).

There are also cognitive hypotheses, such those based on the notion that language processing involves “domain general” cognitive processes that have improved over evolution and also improve during child development (e.g., Saffran et al., 1996; Marcus et al., 1999; Friederici et al., 2011; Perani et al., 2011). This is in contrast to the notion that language involves domain specific modules that have specialized specifically for language processing. In support of domain general hypotheses, there is evidence that the processing of AG structures can engage comparable brain regions as does the processing of natural language material (Friederici et al., 2006; Bahlmann et al., 2008; Folia et al., 2011; Tyler et al., 2011; Petersson et al., 2012). Such hypotheses tend to emphasize the role of the cognitive systems, such as those supporting attention and memory, and how these may have improved during evolution to support language in humans or vocal learning in complex-vocal learners.

## Predictions of motor and other theories, from a modified behavioral perspective

In this final section, we aim to integrate the ideas generated in the previous sections. We first summarize ways in which the prediction from the motor theories could be tested. Second, we summarize predictions from the other theories that we have considered, including cognitive domain-general hypotheses and how the predictions of these theories relate to those from the motor theories. The integration of the experimental and theoretical strands is important for advancing our understanding of language origins and mechanisms, and more generally of animal communication.

### Predictions from motor theories

Strong theories make predictions that can be tested to help to support or refute their different tenets. We suggest that all such theories of spoken language evolution should be tested at both the behavioral and neural levels in order to revise them or to develop better ones. Next we consider the testing of predictions from the different theories in the context of our modified views, based on the accumulating evidence on variability in vocal production and auditory learning abilities in different species.

#### Predictions of motor theories of speech/song perception

These theories suggest that there are considerable benefits for the *perception* of conspecific communication signals in animals that can rely on learned sensory-motor interactions during vocal imitation or vocal learning. Thus, they predict considerable behavioral differences in the perception of learned sounds between so-called vocal learners and non-learners (or even limited-vocal learners). They also predict specialization in at least the sensory-vocal motor interconnectivity in complex-vocal learners that would be lacking or limited in more limited vocal learners. Because limited-vocal learning animals do not readily mimic others' vocalizations and apparently do not have a functional vocal motor forebrain pathway (Feenders et al., 2008), their sensory systems would not benefit from interaction with a forebrain vocal system for the perception of vocal communication signals.

Testing this theory depends on whether results are expected to show absolute differences (presence vs. absence) or differences by degree. Absolute differences between complex- and limited-vocal learners in the behavioral perception of communication signals and the neural substrates that subserve them are unlikely to be found in biological data. Smaller differences could complicate supporting or refuting the theories. For example, dogs have limited vocal modification abilities, but can learn to understand hundreds of human words. Thus some aspects of their auditory behavior and/or neurobiological substrates can be expected to be similar to how humans perceive speech, although dogs certainly lack, at least, the human capacity to comprehend spoken language. The motor theories for speech/song perception are also challenging to test since communication signals acoustically differ in a number of ways between animals and species as does the level of experience that animals have with species-typical communication vs. other sounds.

It is now well known that categorical perception is not unique to humans or to human speech as was originally thought (Ehret, 1987). Another potential challenge to this theory is that human and non-human primates appear to have comparable preferential responses for the processing of voice content in conspecific vocalizations (Belin et al., 2000; Petkov et al., 2008), some aspects of which have now been studied at the neuronal level in monkeys (Perrodin et al., 2011). If, however, the processing of voices that can be imitated in the human brain is subserved by processes that differ from the processes that support voice processing in monkeys, then such findings would likely support the motor theory of speech perception. If, however, these processes are shown to be largely comparable then the results might better support another theory, such as the *motor theory of vocal learning origin*.

#### Predictions of the motor theory of vocal learning origin

This theory underscores a distinction between the vocal *motor pathways* of complex-vocal learners and limited-vocal learners. Unlike the motor theories of speech/song perception, this theory makes no claims about whether the perceptual/learning systems differ between so-called vocal learners and non-learners. This is because the motor theory of vocal learning origin proposes a difference in the forebrain vocal motor pathway in vocal learners and non-learners (Feenders et al., 2008). Behaviorally, this theory is not mutually exclusive with the motor theories of song/speech perception; that is, the tenets of the latter theories can be interpreted to predict that vocal non-learners lack or have a limited access to the forebrain vocal motor pathway for perception. However, the motor theory of vocal learning origin does require that there are little to no differences in the auditory pathway input to the non-vocal motor pathway between vocal non-learners and learners.

Rigorously testing the motor theory of vocal learning origin will be challenging. Ideally one would use genetic/transgenic means to cause the forebrain auditory-motor pathway to duplicate during embryonic development and to form a direct projection from the forebrain to the brainstem motor neurons. Doing so would require discovering the genes that differ in their regulation of the vocal motor pathway or the adjacent non-vocal motor pathways. Candidate genes involved in axonal guidance and neuronal protection are being discovered in both song-learning birds and humans (Matsunaga and Okanoya, 2009; Hara et al., 2012; Horita et al., 2012). The impact on vocal behaviour from the genetic manipulations would need to be evaluated.

One potential challenge to parts of this theory is the observation that some complex-vocal learners (like humans and parrots) can synchronize their movements to a rhythmic beat in music (that is, to dance to a rhythm) whereas no vocal non-learners have been shown to be able to synchronize their movements in this way (Patel et al., 2009a,b; Schachner et al., 2009). The authors of these studies suggested that once the vocal learning pathway evolved, this affected the auditory pathway in such a way that it was differently connected with non-vocal motor pathways in complex-vocal learners. This hypothesis would predict differences in the auditory-motor pathway connectivity of vocal non-learners and learners. Another potential challenge to the motor theory of vocal learning origin is the finding in mice (Arriaga and Jarvis, in press; Arriaga et al., in press) of a limited forebrain vocal motor pathway with a sparse direct projection to brainstem vocal motor neurons, relative to complex-vocal learners. This would not negate the possibility that the vocal learning pathways emerged from a lineage of motor neurons related to the adjacent motor pathway, but it could mean that it might not be necessary to induce brain pathways by duplication. Instead, the vocal learning continuum hypothesis would predict that in complex vocal learners there was independent enhancement of an already existing pathway. In such a situation, the enhancement of the direct projections rather than their presence/absence may be the key difference between complex- and limited-vocal learners.

This theory might be tested with the use of viral vectors containing axonal guidance molecules to strengthen the sparse forebrain to brainstem vocal motor connectivity (e.g., cortico-bulbar projections). Positive outcomes from any such manipulations could be obtained by an animal being able to learn to more flexibly modify its vocalizations. A further possibility is that the innate brainstem vocal-production pathway may be able to separately support limited-vocal learning, such as, the pitch matching seen in mice and marmosets.

### Predictions from gestural theories

#### Predictions of the gestural theory of spoken-language origin

The predictions of this theory are similar to the *motor theory of vocal learning origin* in that both predict a comparable perceptual system but differences in the production systems of complex-vocal learners. This theory goes a step further to hypothesize that brain pathways used to produce speech are intertwined with pathways used to perform learned gestures. Interestingly, Taglialatela and colleagues have observed Positron Emission Tomography (PET) activations in the chimpanzee inferior frontal cortex that occur after vocal production and gesturing but not after gesturing alone (Taglialatela et al., 2011). To more thoroughly test this theory, one would need to determine if the neurons and connectivity for gesturing (such as controlling hand movements) are the same as those for vocal production or form mixed neuronal subpopulations. Since, apes are a protected group, to study such neuronal populations might require developing a different non-human primate model system that can both gesture and vocalize, if possible. The gestural theory also relies on there being a forebrain motor cortical region that “controls” the sequencing of vocalizations and gestures in humans. Whether such a region would be found in the brains of non-human primates is uncertain. A critical test might require inactivating the currently poorly understood regions for vocal control and gesturing in certain non-human primates, and finding whether one set of behaviors can be maintained without the other. Efforts such as these could also help to clarify to what extent the motor theory of vocal learning origin depends on a gestural motor system.

#### Predictions of the gestural “mirror neuron” theory of language evolution

This theory predicts a lack of or weakness in the auditory-vocal motor link in limited-vocal learners or vocal non-learners. In other words, mirror neurons are engaged in human and non-human primates for gestural and other sensory-motor tasks but are not used in non-human primates for vocalization. One might predict that the mirror-neuron pathway for vocalization is: (1) not available for vocal production and imitation in limited-vocal learners such as chimpanzees, monkeys, many birds, etc.; and/or (2) that it is generally available for motor production in many animals but does not directly engage the auditory pathway or the pathway for innate vocal production. Given that to date linked auditory activated vocal mirror-neurons have only been reported in songbirds (Prather et al., 2008), a number of interesting issues remain to be tested across the species. For instance, are there auditory-vocal mirror-neurons engaged in vocal behavior in any of the limited-vocal learning birds, non-human primates or other vertebrates? The comparative connectivity data on the origins of the “arcuate fasciculus”—the classical language-related tract that links fronto-temporal brain regions in humans—remain controversial (Frey et al., 2008; Rilling et al., 2008) and do not seem able to currently provide strong evidence in support of or against a “weakness” in the auditory-vocal motor pathway in so-called vocal non-learners. Nevertheless, one way to functionally test whether the mirror neuron hypothesis is a viable mechanism for motor imitation would be to reversibly inactivate the so-called sensory-motor mirror neurons and determine if this affects vocal or other motor learning. This could be done with non-invasive trans-cranial magnetic stimulation in humans during speech processing, neuropharmacological inactivation in song-learning birds during song processing, or during some aspects of auditory/visual processing in any species with such neurons.

### Predictions from a “sensorimotor integration” model

The “sensorimotor integration” model—of efference copies from prefrontal and premotor regions during speech production that modifies auditory processing and the perception of sounds—emphasizes the commonalities across the species regarding sensorimotor interactions (Rauschecker and Scott, 2009; Rauschecker, 2011). It thus differs in key ways from the cross species differences predicted by the motor theories of speech/song perception. This model could be largely compatible with the *motor theory of vocal learning origin* (section “Predictions of the Motor Theory of Vocal Learning Origin” above) if, as the motor theory proposes, the key difference between complex-vocal learners and limited-vocal learners is not in the perceptual system where sensori-motor interactions can help but in the form of the forebrain motor subsystem that is engaged. The model as proposed by Fee in songbirds is more applicable to sensorimotor pathways generally, suggesting that the song learning system has integrated with the adjacent motor pathways (Fee, 2012). Such a model is also consistent with the *motor theory of vocal learning origin*. Neuroimaging and neurophysiological data from multiple brain regions will be required to better evaluate the efference effects from frontal to auditory regions in complex- and limited-vocal learners. Results showing that the efference signal for vocal or other behavior differs in humans relative to non-human animals might better support the *motor theory of speech/song perception*. For further details on this model in humans, its historical basis and testable predictions see (Rauschecker and Scott, 2009; Rauschecker, 2011).

### Predictions from cognitive evolution hypotheses: evolutionary neuroscience of syntactic-related processes

Cognitive hypotheses consider that complex-vocal learners, and more specifically humans, have enhanced capacities in cognitive systems broadly (e.g., learning, memory, attention, etc.) to support enhanced learned vocal communication perception or production. This is in contrast to notions that there are neurobiological substrates specifically dedicated to support speech/song capabilities. We discuss predictions of such hypotheses in four contexts: “domain” general and language specific predictions, and predictions revolving around primate and bird models.

#### General predictions from hypotheses on the evolution of cognitive systems

Behavioral predictions are that all animals can show varying levels of sensory or vocal learning, but are limited primarily by their cognitive abilities. Neurobiologically, limited-vocal learners would have the functionality of these systems with reduced capacity. The notion that most cognitive systems are improved in capacity in complex-vocal learners predicts a testable correlation between cognitive capacity and the level of engagement of cognitive processes (e.g., in learning increasingly more complex artificial-grammar sequence structures).

General support for cognitive evolution hypotheses are as follows. Some authors have obtained data that suggests that unlike humans, monkeys have reduced capacity for auditory recognition memory and may not directly engage the hippocampal memory circuit for auditory recognition memory (Fritz et al., 2005; Munoz-Lopez et al., 2010). In this regard, it remains to be determined whether human or songbird auditory recognition memory is indeed better than in non-human primates (or other limited-vocal learners), or if humans benefit from being able to semantically label speech to gain direct access to long-term memory circuits. The finding that only vocal learners can synchronize to a rhythm (Patel et al., 2009a) can support hypotheses on the evolution of cognitive systems. In this case, non-linguistic behavioral traits might be shown to depend on substrates that improved alongside or after the evolution of vocal learning. Additionally, there are various sorts of data, including anecdotal evidence, that some vocal learners (parrots, corvid songbirds, dolphins, elephants) have more complex cognitive behaviors relative to other animals that are more closely evolutionarily related to humans (Emery and Clayton, 2009). At a genetic level, it was recently discovered that humans have several unique duplications of the gene SRGAP2 (the SRGAP2 gene codes for SLIT-ROBO Rho GTPase-activating protein 2) that controls neural connectivity not found in any other primate or mammalian species tested to date (Charrier et al., 2012; Dennis et al., 2012); the extra copies when placed in mice induce an increase in dendritic spines and longer lasting spine immaturity, as is seen in human brains. The unique human gene duplications of SRGAP2 are hypothesized to be associated with greater learning capacity.

Additional comparative testing of behavioral abilities and of neurobiological, and genetic substrates is needed to provide stronger evidence either in favor of the evolution of cognitive “domain general” systems or alternatively in favor of “domain-specific” substrates that have considerably specialized for vocal production learning in humans or other vocal learners. Such testing will require two types of comparisons: (1) determining whether there are considerable vocal “domain specific” specializations not used for non-vocal learning capabilities (i.e., specializations in the auditory learning and vocal motor pathways); and (2) determining whether the auditory and vocal learning pathways in the brains of humans and other *vocal learners* function at a higher level of complexity than related brain pathways in other species. This would involve comparative analysis of cognitive and both auditory/vocal and non-auditory/non-vocal processing demands.

An interesting way forward for testing the tenets of various theories is with AGL and/or “statistical learning” paradigms, which are well suited for study in both humans and non-human animals. Neurobiological substrates for AGL and statistical learning can be evaluated in relation to language-related processes in the human brain. Such approaches seem well suited to test hypotheses on at least the evolution of cognitive systems and clarify the presence of domain general or domain specific substrates.

#### Syntactic complexity and the neurobiology of human language

Neuroimaging and neuropsychological work in humans has highlighted that how the human brain network for syntactic learning is engaged depends on the sequencing demands and types of structural relationships being evaluated (i.e., “syntactic complexity,” Hagoort, 2009; Friederici, 2011; Fitch and Friederici, 2012; Petersson et al., 2012). From such results, a set of evolutionary “syntactic complexity” hypotheses have emerged (e.g., Friederici, 2004, 2011; Hurford, 2012; Petkov and Wilson, 2012). These propose that ancestral communication systems may have faced evolutionary pressures to manage greater sequencing demands in sensory input and/or motor output. This may have led to the evolution of enhanced systems in the human brain for processing syntactic complexity and the capacity for these to be learned during development (Friederici et al., 2011; Perani et al., 2011).

To bring this together in a model, Figure 5A schematizes the human auditory system engaged in syntactic perceptual learning, focusing, for brevity, on pathways interconnecting temporal and frontal cortical regions (for further details see, e.g., Friederici, 2011). Some of the model is based on the evidence in humans for brain regions and networks that are either sensitive to the violation of different types of learned AG structures (e.g., Friederici et al., 2006; Bahlmann et al., 2008, 2009; Friederici, 2009; Hagoort, 2009; Makuuchi et al., 2009; Petersson et al., 2012) and/or engaged in processing natural language structure (Marslen-Wilson and Tyler, 2007; Friederici, 2011; Tyler et al., 2011; Petersson et al., 2012). Figure 5B shows how the syntactic perceptual learning system is thought to engage with the vocal production learning system. These pathways are thought to explain different levels of complexity in AGL (Friederici, 2004; Friederici et al., 2006; Bahlmann et al., 2008; Friederici, 2011). For example, when humans build the initial syntactic structural analysis (such as evaluating only adjacent-relationships in a FSG), regions such as the frontal operculum (FOP) in the inferior prefrontal or insular cortex are engaged. The FOP interconnects with the anterior temporal lobe via the ventral uncinate fasciculus (UF) pathway (Figure 5A) (Friederici et al., 2006). However, when humans evaluate grammatical structures based on more distant non-adjacent relationships in FSG (Friederici, 2004; Petersson et al., 2012) or those having nested relationships (Bahlmann et al., 2008), then at least Brodmann area 44 (BA 44, a part of Broca's territory in the inferior frontal gyrus) is engaged in evaluating grammaticality (Friederici et al., 2006; Bahlmann et al., 2008; Folia et al., 2011; Petersson et al., 2012). BA44 is interconnected with the posterior temporal lobe via the dorsal superior-longitudinal fasciculus (SLF, Figure 5B), which includes parts of the human arcuate fasciculus. In humans, it is hypothesized that a different ventral (extreme capsule) pathway supports greater demands in syntactic complexity or syntactic-semantic relationships (Friederici, 2011). Unlike the ventral UF pathway interconnecting the FOP with the anterior temporal lobe, this other ventral pathway interconnects BA 45 and anterior temporal lobe regions (Figure 5A in orange, next to the FOP pathway in red) (Friederici, 2011). This model is similar to the dual pathways (dorsal and ventral streams) model for auditory and language processing, although there are also some differences (for reviews see: Rauschecker and Scott, 2009; Friederici, 2011; Rauschecker, 2011).

#### Non-human primate hypotheses: multiple pathways for “proto-syntactic” learning

Friederici (2004) proposed neurobiological substrates that in non-human primates might have been evolutionary substrates for proto-syntactic learning in humans. Petkov and Wilson (2012) extended this prediction into several subhypotheses, based on the finding that tamarin monkeys (Fitch and Hauser, 2004) and macaques (Wilson et al., 2011) can learn adjacent dependencies in an auditory AG with sequences that only require FSG processes. These are that: (1) the ventral, UF pathway (involving the monkey anatomical homologs of the FOP such as areas vF4/vF5 and parts of the inferior frontal insula) is engaged in the processing of adjacent relationships in AGs; and, (2) the dorsal, superior-longitudinal fasciculus pathway (including BA 44) is engaged for evaluating greater complexity in FSGs, such as non-adjacent relationships (Petersson et al., 2012), if the monkeys can learn these (Figure 5C). For further specifics and alternatives see: (Wilson et al., 2011).

#### Bird hypotheses: multiple pathways in songbirds for “syntactic-like” sensory learning

Regarding brain regions that might support songbird AGL, the study in Bengalese finches by Abe and Watanabe (2011) showed that expression of the immediate early gene *egr*1 around the lateral magnocellular song nucleus of the anterior nidopallium (LMAN) was associated with whether these finches could learn aspects of AG sequences. However, it remains unclear whether these results relate to the AG structure or acoustical cues that were present in the “violation” sequences that were used for testing (see critique by: Berwick et al., 2011; Ten Cate and Okanoya, 2012). Another issue is that the areas studied in Bengalese finches, around LMAN, belong to the same nidopallial region that Feenders et al. (2008) found movement-driven (hopping) gene expression. Thus, it is not clear if the activation that was seen here could have resulted from differential movements of the animals to the different testing conditions or an association of the movement task with the grammatical processing. In all cases, we would hypothesize that some parts of the bird auditory system (in both complex-vocal learners and potentially also other birds) engages the motor areas adjacent to the song nuclei in evaluating AG structural relationships and to prepare non-vocal motor responses (Figure 5D). If so, a question that arises is in which neurobiological substrates would complex-vocal learners differ from more limited vocal learning birds or non-human primates? Possibly complex-vocal learners might be able to learn higher complexity in AG structures and for this potentially engage some parts of the vocal learning nuclei that would be unavailable to the limited vocal learners.

#### Relationship to predictions from motor and other theories

The hypotheses of Figures 5C,D do not illustrate the possible greater or lesser reliance on subcortical structures (such as the basal ganglia and thalamus) and/or cerebellum to support the learning of AG sequences. Some of these structures form a part of the system for motor-related learning and thus would link to and/or help to address predictions of motor theories. Moreover, how the animals learn AGs needs to be more carefully considered since we would expect different neurobiological substrates to be engaged if, for instance, the animals are engaged in implicit learning (e.g., habituated to grammatical sequences prior to testing) or are trained to discriminate grammatical vs. ungrammatical sequences, which would engage reward-dependent pathways (Petkov and Wilson, 2012). The predictions that we make in Figure 5, if supported could challenge the motor theory of speech perception/production that proposes considerable differences in the perceptual systems. Such results could instead support the motor theory of vocal learning origins. However, the hypotheses in this section cannot clarify whether the systems would depend on the gestural system, if no gestural or motor imitation component is involved in the experimental design. Moreover, the extent to which the systems in Figure 5 differ across the species could also be used to test the sensori-motor integration or domain general hypotheses.

A major limitation in testing motor and other theories is that relatively much less effort has been made to study the basic behavioral phenotypes and underlying neural pathways that control either auditory-vocal or non-vocal pathways in vocal non-learning animals. As we have considered, such data can provide crucial insights on spoken language origins when compared with data from humans and complex-vocal learners, in which there has been a considerably greater focus. Thus, to validate or falsify the different hypotheses and to generate new ones, a much greater amount of additional comparative work is needed and any “one animal centric approaches” cannot be encouraged. We all tend to emphasize the literature and work in our own study groups or in a limited few species, but it remains important for researchers to continue to look beyond their immediate species of study.

## Conclusions

This review has considered the behavioral and neurobiological data in complex-vocal learners such as, humans and songbirds and how they relate to data from so-called “vocal non-learners.” We noted that the evidence provided by several recent examples in the animal behavioral literature motivates a revision of the hypothesized “vocal learning” vs. “vocal non-learning” distinction. We outlined an alternative hypothesis of greater variability in vocal learning categories and in a related but different behavioral phenotype, namely, auditory (sensory) sequence learning. Upon this modified perspective of the behavioral literature, we considered neurobiological distinctions between “vocal learners” and “non-learners,” questioning whether these distinctions will be useful for clarifying behavioral results or whether behavioral variability can help us to understand the neurobiological substrates and distinctions at a finer level.

Motor, gestural and other theories were considered and predictions made, including from the perspective of animals that are not complex-vocal learners. We also considered the distinctions between the tenets of a number of theories regarding the sensory learning (reception) and motor (production) systems, including interconnectivity and interactions with the neurobiological systems supporting cognitive processes and how these may have evolved. This was used to make specific predictions, exemplified in humans, birds, and non-human primates to investigate the neurobiological substrates that might be evolutionarily related, either by common descent or convergence, to the ones that humans rely on for language.

Taking all of this into consideration, we highlighted the need for comparative approaches that more closely consider the behavioral and neurobiological data on sensory learning and vocal production abilities in many vertebrates. This will be critical for staying objective, empirically grounded, and realistic with regards to the aspects of human spoken language that different animal species could address or serve to model. We hope that this paper has been able to underscore the importance of and to encourage further interdisciplinary cross-species work for clarifying not only the origins of spoken language but also the conserved components and specializations present in the neurobiological systems supporting the communication abilities of animals.

### Conflict of interest statement

The authors declare that the research was conducted in the absence of any commercial or financial relationships that could be construed as a potential conflict of interest.

## Footnotes

^1^It is interesting that in some songbirds, only the males learn to sing. In these species, the females are born with song nuclei, but these atrophy as the females become adults, constituting a developmental loss of the trait. Delivery of high levels of estrogen in nestlings can prevent the atrophy of the song learning nuclei in these females (Konishi and Akutagawa, 1988). Hormonal manipulations in “vocal non-learners” do not seem to reveal a suppressed vocal system.
